# Effects of Temperature on the Fretting Wear Behavior of 2.25Cr-1Mo Tubes against Gr5C12 Rods

**DOI:** 10.3390/ma13153388

**Published:** 2020-07-31

**Authors:** Xu-Dong Chen, Li-Wen Wang, Ling-Yun Yang, Rui Tang, Zhen-Bing Cai

**Affiliations:** 1 Tribology Research Institute, Key Lab of Advanced Technologies of Materials, Ministry of Education, South West Jiaotong University, Chengdu 610031, China; cxdong6758@my.swjtu.edu.cn (X.-D.C.); ylyun@my.swjtu.edu.cn (L.-Y.Y.); tr358@my.swjtu.edu.cn (R.T.); 2 Central Research Academy, Dongfang Electric Corporation, Chengdu 611731, China

**Keywords:** sodium-cooled fast reactor, heat transfer tube, 2.25Cr-1Mo, fretting wear, temperature changes, wear mechanism

## Abstract

In the heat exchangers of sodium-cooled fast reactors, sodium flow can cause the tubes to vibrate, resulting in fretting wear damage due to the contact between the tubes (2.25Cr-1Mo steel) and their support plate (Gr5C12 alloy). In this work, the effects of temperature on the fretting wear behavior of a 2.25Cr-1Mo heat transfer tube on a Gr5C12 alloy rod were studied. The results showed that the coefficient of friction (COF) and wear volume increased first and then decreased with the increase in temperature. Moreover, 2.25Cr-1Mo showed great wear performance at high temperatures than at room temperature and 80 °C, because of the antifriction nature of the oxidative layer and the high hardness of the tribological transformed structure layer. As the temperature increased, material transfer and plastic deformation became increasingly obvious, but average wear depth decreased. This provides data support for the practical engineering application of 2.25Cr-1Mo steel at elevated temperatures. Wear mechanisms were found to depend modestly on temperature and largely on normal load. As temperature increases, the wear mechanism gradually changes from abrasive wear to adhesive wear.

## 1. Introduction

Current nuclear reactors have progressed to the fourth generation [1]. Sodium-cooled fast reactors are the focus of research on fourth-generation nuclear reactors [2]. These reactors use sodium metal as a coolant. Sodium is characterized as having a violent chemical reaction with water, water vapor, or oxygen [3]. When the tube of a heat exchanger leaks or breaks, a severe exothermic reaction occurs between sodium and water. This sodium–water reaction event is generally considered in the design of sodium-cooled fast reactors [4]. Therefore, studying the damage mechanism of heat transfer tubes is necessary. The literature indicates that fretting wear due to the flow-induced vibration (FIV) [5] between the structure and fluid in a heat exchanger is one of the damage forms of heat transfer tubes. Fretting wear is also described as a type of wear phenomenon that occurs on two contact surfaces subject to small amplitude oscillations [6].

Current studies on heat transfer tubes are mainly focused on the second generation of pressurized water reactors. The 690 alloy was widely used in heat transfer tubes in the second generation of pressurized water reactors due to its excellent intergranular corrosion and intergranular stress corrosion cracking properties [7]. In recent years, fretting wear research on Inconel 690 alloys has covered contact forms [8,9], environment and temperatures [10,11,12], loads, and displacements [13,14,15,16]. In addition, a number of studies have focused on the effects of grain size and hardness [17], carbide size, and spacing [18] on the fretting wear properties of Inconel 690. Mi et al. [19] found that, at 90 °C, the wear mechanisms are delamination and abrasive wear. At 200 °C and 285 °C, the wear mechanisms are delamination, adhesion, and abrasive wear. Lu et al. [20] characterized the wear surface and subsurface layer of Inconel 690 alloys and found five layers, namely, an oxide layer, mixed layer, tribological transformed structure (TTS) layer, plastic deformation layer, and base material.

The low-alloy ferritic bainite steel, 2.25Cr-1Mo steel, is a candidate material for the high-temperature heat transfer tubes of fourth-generation nuclear reactors [21]. For the past 30 years, scholars have carried out extensive studies on the microstructure [22], high-temperature creep [23,24], welding [25,26], and radiation resistance [27] of 2.25Cr-1Mo steel. However, studies on the wear properties of 2.25Cr-1Mo steel are relatively few. Pattnaik et al. [28] systematically studied the erosion and wear of 2.25Cr-1Mo steel. Their results showed that, under normal conditions, impact speed is an important factor that causes maximum wear. Under thermal aging conditions, erodent size is the most important factor affecting wear. A similar study was carried out by Sahoo et al. [29]. The results indicated that the Larson–Miller parameter exerts a great static influence of 46.33%, impingement angle has an influence of 42.51%, impact velocity has an influence of 7.47%, and the erodent size has an influence of 1.13% on the solid particle erosion behavior of this alloy.

Therefore, the current work mainly studies the tangential fretting wear properties of 2.25Cr-1Mo steel at varied temperatures. This study is significant in the evaluation of the wear performance of 2.25Cr-1Mo steel.

## 2. Experimental Procedure

### 2.1. Materials and Specimens

In this work, 2.25Cr-1Mo steel was investigated under dry sliding. The material of its friction pair was a Gr5C12 alloy rod, which was used as the tube support plate material in the heat exchangers. All test materials were supported by the Central Research Academy of Dongfang Electric Corporation (Chengdu, China). The chemical composition (wt.%) is listed in Table 1. The original tube was cut with an electric spark wire. The length of the 2.25Cr-1Mo steel tube specimen was 30.0 mm, and the outer and inner diameters were 16.0 and 11.0 mm, respectively. The original plate was cut into strips with an electric spark wire, then machined into rods (with a diameter of 10.7 mm). Finally, the Gr5C12 rod was cut to a specimen with a length of 20.0 mm. All samples were sanded mechanically, and polished by a polishing machine. So, the surface roughness of all tested materials was 0.4 μm. The samples were ultrasonically cleaned with banana oil, deionized water, and absolute ethanol and dried with hot compressed air before the tests.

### 2.2. Tribological Testing and Analysis

**Figure 1:**
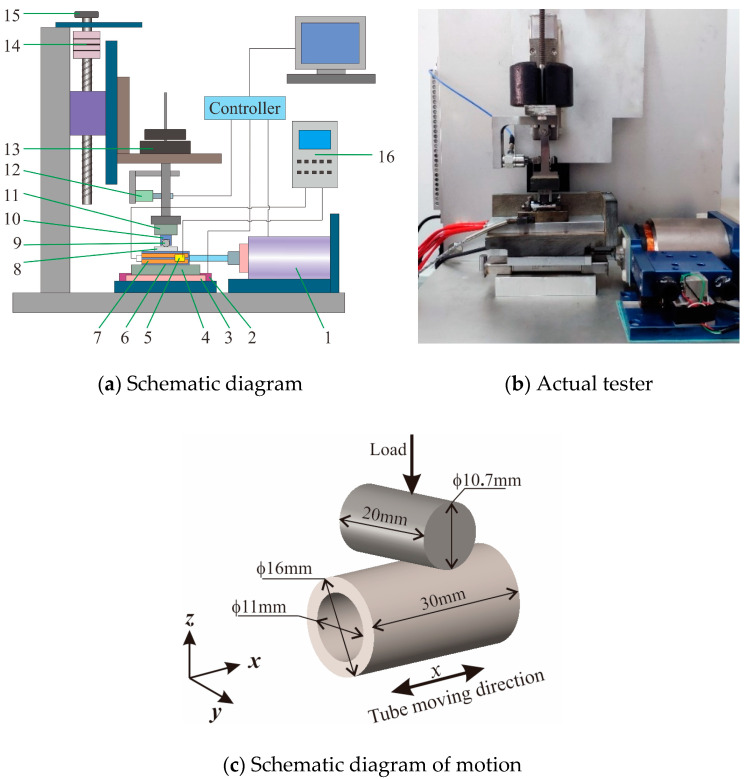
Test equipment (**a**,**b**): (1) voice coil motor; (2) grating ruler; (3) guide rail; (4), (11) heat insulation plate; (5) temperature sensor; (6) lower fixture; (7) heating rod; (8) lower sample; (9) upper sample; (10) upper fixture; (12) force sensor; (13) weights; (14) envelope; (15) spinner handle; (16) temperature control box, and schematic diagram of motion (**c**).

All the tests were performed using self-developed high-temperature tangential fretting wear equipment, which was mainly composed of a voice coil motor, frame and weight lifting platform, control and measurement systems, data acquisition systems, and other spare parts. The device was appropriately designed and easy to use. A simple schematic diagram is drawn in Figure 1a. The control and data acquisition programs were installed on a computer. After clamping the sample, the computer was used to input the test parameters (amplitude and frequency). The general procedure of the test was as follows. In the first step, the lower sample (8) and the heating rod (7) were fixed and inserted into the lower fixture (6). The lower fixture (6) and heat insulation plate (4) were fixed onto the guide rail (3) by bolts. In the second step, the upper sample (9) was fixed on the upper fixture (10), and the upper fixture (10) and heat insulation plate (11) were fixed onto the equipment together. In the third step, the temperature control box (16) was used to control the heating rod (7) for heat generation until the temperature sensor (5) returned a temperature that reached a preset value. In the fourth step, the spinner handle (15) was used to drop the horizontal position of the upper sample (9) until it simply made contact with the lower sample (8). Then, a weight (13) was used to press the upper sample (9) against the lower sample (8). In the fifth step, the voice coil motor (1) was controlled by the computer to start reciprocation (at *x* direction). The grating ruler (2) and force sensor (12) monitored the displacements and friction signal during the test in real time and cyclically fed them back to the computer. The image of the actual equipment is shown in Figure 1b and the schematic diagram of motion is shown in Figure 1c.

Table 2 shows the specific experimental parameters. Given the influence of a high temperature environment on the fretting wear properties of 2.25Cr-1Mo steel, four temperature gradients (room temperature (RT), 80 °C, 225 °C, and 450 °C) were set. Two types of normal loads (10 N, 20 N) (close to actual working conditions), displacement amplitudes (50 μm, 100 μm), and a frequency of 5 Hz and 10^4^ cycles were selected. Tests 1–3 (20 N-50 μm, 10 N-50 μm, 10 N-100 μm) were designed at all temperatures, and each group of experiments was repeated three times. After the tests, the three sets of repeated test data were averaged and the corresponding error bar was calculated (using the standard deviation). The surface morphology of the worn scar was observed using a super depth field microscope (VHX-7000, KEYENCE, Osaka, Japan). A white light interference microscope uses the principle of interference to measure the optical path difference to determine the relevant physical quantities. Therefore, it can be used to determine the three-dimensional shape of the wear scar, and then the wear area, depth, and volume can be obtained by the analysis software Vision. The detailed morphology and chemical reaction of the worn scars were studied by scanning electron microscopy (SEM, JSM 7800F, JEOL, Tokyo, Japan) and energy dispersive X-ray spectroscopy (EDX, Aztec X-Max 80, Oxford instrument, Abingdon, UK), respectively. In addition, electron probe micro-analyzer (EPMA, JXA-8230, JEOL, Tokyo, Japan) spectroscopy was conducted to examine the main elements of the cross section of the worn scar.

## 3. Results and Discussion

### 3.1. Friction Coefficient

**Figure 2:**
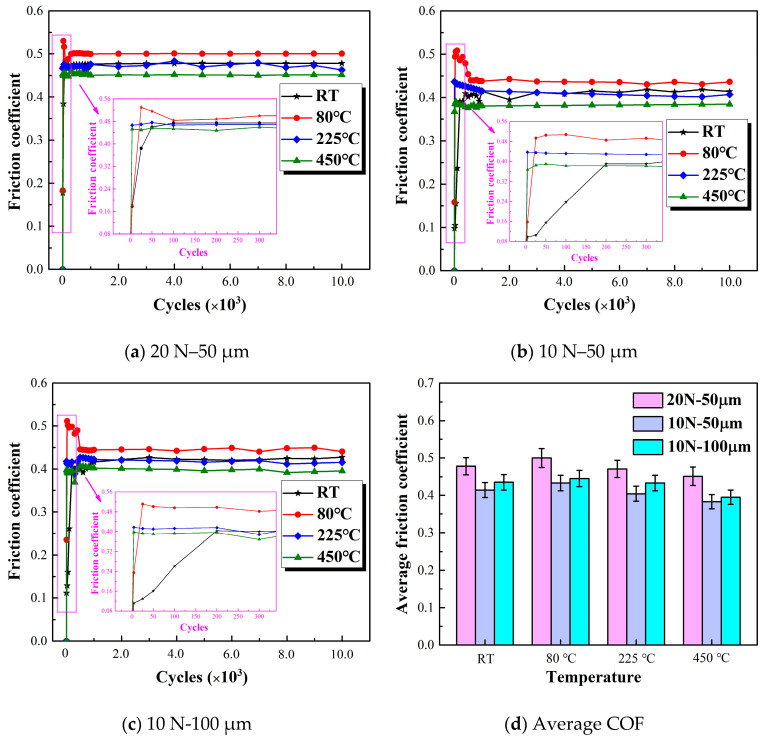
Evolution of COF with the number of cycles at varied temperatures.

The coefficient of friction is the result of dividing the average friction force (using 10% of the collected data near the peak friction force) by the normal load. The evolution of the coefficient of friction as a function of cycle numbers is shown in Figure 2. In general, as the number of cycles increases, the COF can be approximately divided into five stages (initial, ascending, peak, descending, and steady stages) [4]. The initial and ascending stages generally involve the removal of the oxidative layer of the material [30]. At 225 °C and 450 °C, the COF reaches the steady stages only after a few tens of cycles. This behavior indicates that the initial oxidative layer of 2.25Cr-1Mo steel is easily removed at high temperatures. The generation and removal of abrasive debris also reach a dynamic balance faster in high temperatures than in low temperatures. In nearly all the tests, as the temperature increases, the average COF increases first and then decreases (Figure 2d). This result is consistent with Mi’s research on 690 tubes in high-temperature water [19]. The maximum average COF in the stable stage is 0.500 at 80 °C, and the minimum COF is 0.451 in the stable stage at 450 °C (Figure 2d). The COF also increases with the increase in load and amplitude [16,19].

**Figure 3:**
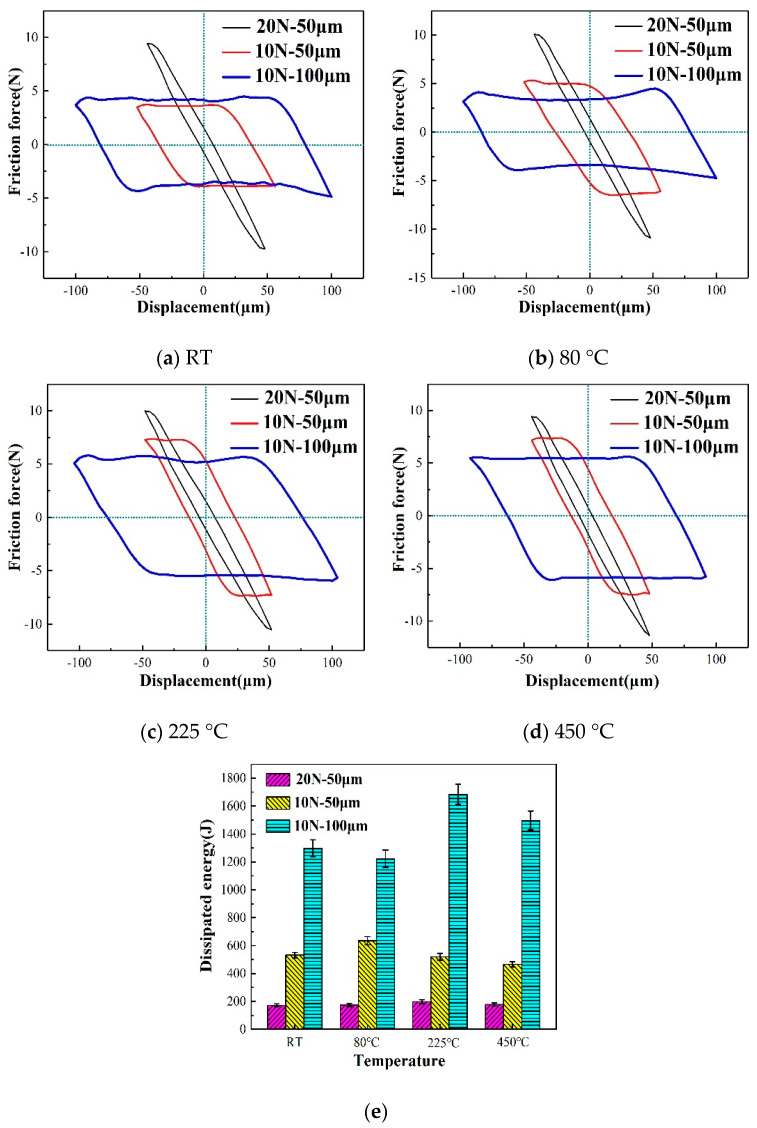
Ft–D curves (**a**–**d**) and dissipated energy (**e**) with varied temperatures, cycles = 10^4^.

The curve of friction force and displacement (Ft-D) between contact surfaces is the most basic and important indicator of tangential fretting wear [31]. The fretting maps of the three groups of tests after 10^4^ cycles are shown in Figure 3. In general, temperature exerts some influence on the fretting model, but greater influence comes from the normal load and sliding displacement [16,32]. At 20 N, the fretting map is linear at room temperature or a high temperature, indicating that the fretting model is a partial slip condition. However, at 10 N, the fretting map is basically a parallelogram, implying that the fretting model is a gross slip condition. As the amplitude increases, the gross slip becomes pronounced. As shown in Figure 3c,d, the fretting maps at 225 °C and 450 °C, shown with a red line, show an oval shape. This condition indicates that the slip regime becomes a mixed regime with the increase in temperature. Meanwhile, the dissipated energy within Figure 3a–d was calculated (Figure 3e). For a single cycle, the dissipated energy is the area within a hysteresis loop between tangential force and relative slip (Ft–D plot) [33]. The results show that the dissipated energy has a negative correlation with the load and a positive correlation with the amplitude [34]. However, there is no clear relationship between the temperature and dissipated energy.

### 3.2. Wear Mechanisms

**Figure 4:**
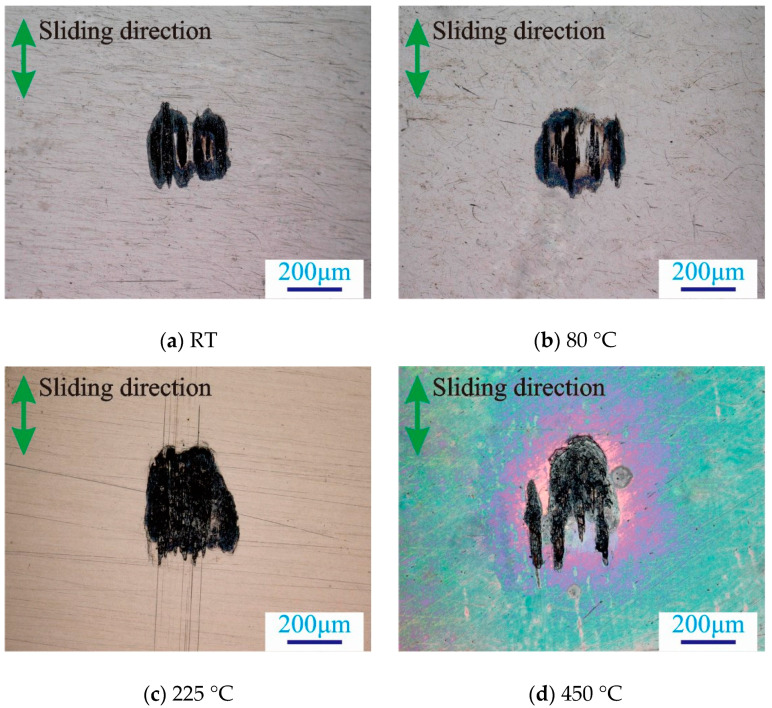
Super depth field micrographs of worn scars at varied temperatures: F = 20 N, D = 50 μm.

The fretting wear property of the 2.25Cr-1Mo steel tube was analyzed, and the worn scars were observed, as shown in Figure 4. When the temperature increases, the surface color of the sample changes first. Especially at 225 °C and 450 °C, the color of the sample surface is clearly different. Meanwhile, the black oxide on the surface of the worn scar increases gradually with the temperature, thereby indicating that the severe oxidation of the heat transfer tubes occurs at a high temperature. The edge of the worn scar is more severely worn than the middle of the worn scar (Figure 4).

**Figure 5:**
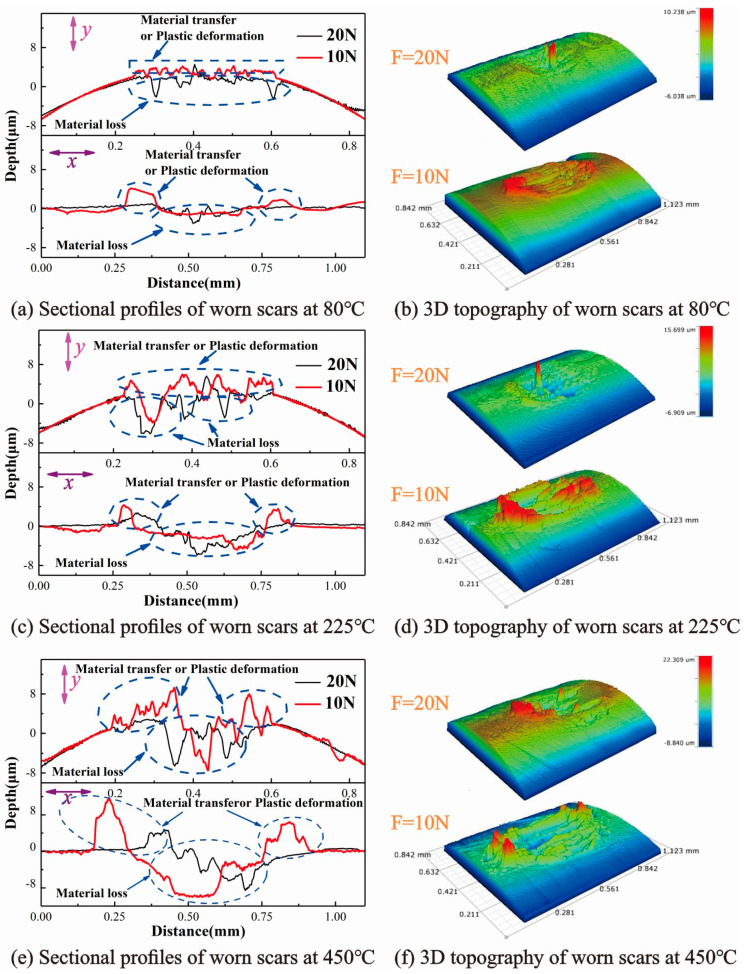
Sectional profiles and 3D topography of worn scars under different normal loads, D = 50 μm.

For further study of the worn scars, Figure 5 presents the sectional profiles and 3D topography of the worn scars. In the direction perpendicular to the sliding direction (*y* direction), the profile of the black line is high in the middle and low on both sides, while the profile of the red line is high on both sides and low in the middle [35]. In the direction parallel to the sliding direction (*x* direction), the profile of the red line is significantly raised at both ends, while the profile of the black line is relatively smooth (Figure 5a). Meanwhile, the deeper material loss, material transfer, or plastic deformation increases with temperature (Figure 5a,c,e), indicating that high temperatures (≥225 °C) may cause deeper material wear.

At 10 N, the edge area of the worn scars is significantly high, especially in the edge area parallel to the sliding direction (Figure 5b). However, at 20 N, the edge area of the worn scar is relatively flat, and the middle area is high. These features also prove that gross slip occurs at 10 N, and partial slip occurs at 20 N. Moreover, the existence of furrows can be clearly seen in Figure 5b,d,f, which are the traces left after the material is peeled out of the friction interface [19]. The red mountain peak increases with the temperature, thereby indicating that high temperatures (≥225 °C) may cause severe material transfer or plastic deformation.

**Figure 6:**
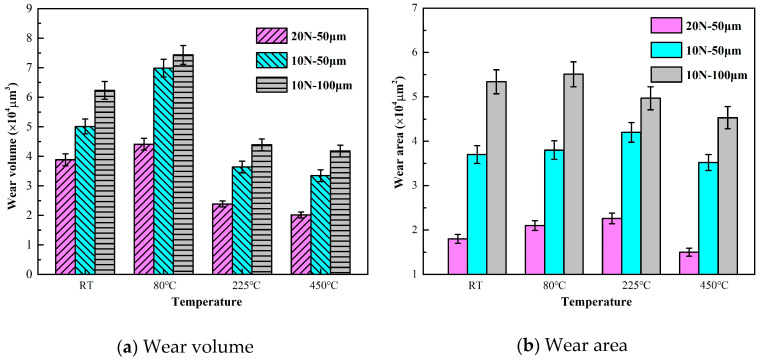
Wear volume and wear area of 2.25 Cr-1Mo steel.

The worn scars were quantitatively studied further. Figure 6 shows the results of wear volume and wear area. When the temperature increases, the wear volume first increases and then decreases. The maximum wear volume occurs at 80 °C. By contrast, the wear volume is small at 225 °C and 450 °C and presents a minimal difference under the same test parameters. Thus, deeper material wear does not mean maximum material removal. At the same temperature, the wear volume increases with displacement and decreases with an increase in load. Thus, wear volume is greatly affected by temperature, normal load, and sliding displacements. Meanwhile, the wear area increases first and then decreases with an increase in temperature, but the change is not obvious. The maximum wear area for tests 1 and 2 occurs at 225 °C, whereas the maximum wear area for test 3 appears at 80 °C. Temperature exerts a slight effect on wear area, and load and amplitude are the main factors affecting wear area.

**Figure 7:**
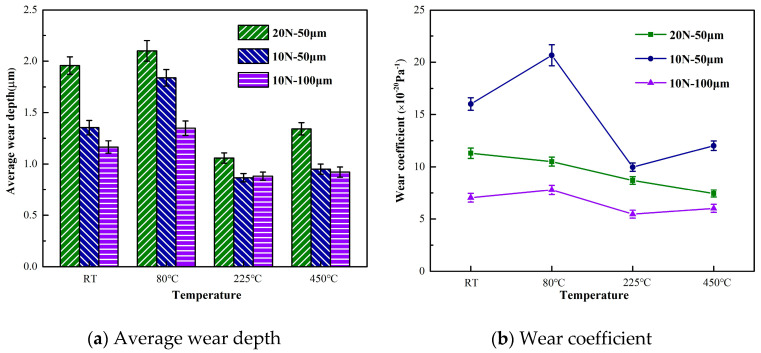
Average wear depth and wear coefficient of 2.25 Cr-1Mo steel.

Figure 7 shows the results of the average wear depth and wear coefficient. The average wear depth is obtained by dividing the wear volume and wear area. As shown in Figure 7a, all the average wear depths of the three groups of tests are larger at room temperature and 80 °C than at 225 °C and 450 °C. In other words, 2.25Cr-1Mo steel has localized deep wear at high temperatures, but the average depth of the entire wear scar is lower than that at normal temperatures. In combination with the wear volume in Figure 6, the material shows reduced spalling at 225 °C and 450 °C, thereby indicating that high temperatures may protect materials. This result may be attributed to the antifriction nature and stabilized high lubrication properties of the oxidative layer and the effect of plastic deformation [20,36].

The formula for calculating the wear coefficient has been described in the following form [37]:

(1) $$\frac{dD\left(x\right)}{dN}=K\times q\left(x\right)\times \left(4\delta \right)$$

where *D*(*x*) and *q*(*x*) are the wear depth and contact shear force, respectively, *N* is the number of fretting cycles, δ is the displacement amplitude, and *K* is termed as a wear coefficient with the unit of Pa^−1^. If we focus on the average depth, Equation (1) is written as

(2) $$\frac{dD_{ave}}{dN}=K\times q_{ave}\times \left(4\delta \right)$$

where $D_{ave}$ is the average wear depth and $q_{ave}$ is the average shear traction (friction force). The wear coefficient is inversely proportional to the normal load and sliding displacement. The statistical results of the wear coefficient in Figure 7b support this result. The wear coefficients of tests 2 and 3 first increase and then decrease with an increase in temperature. The wear coefficient of test 1 decreases with increases in temperature. This phenomenon shows that temperature has less influence on the wear coefficient than normal load and sliding displacement. The value of the wear coefficient reflects the degree of wear in a single cycle. Therefore, each fretting cycle of test 2 achieves the greatest wear to the 2.25Cr-1Mo steel tubes, and the wear of each fretting cycle of test 3 is minimal.

**Figure 8:**
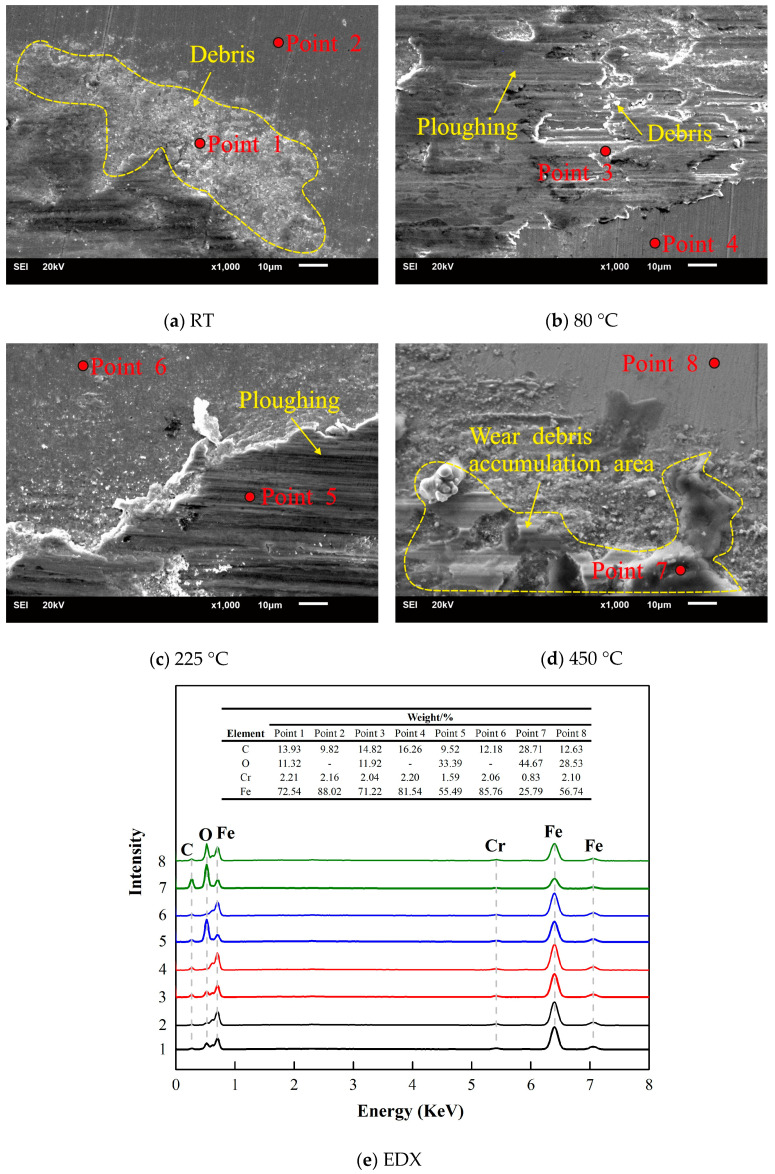
SEM micrographs and EDX of worn scars, D = 50 μm, F = 10 N.

**Figure 9:**
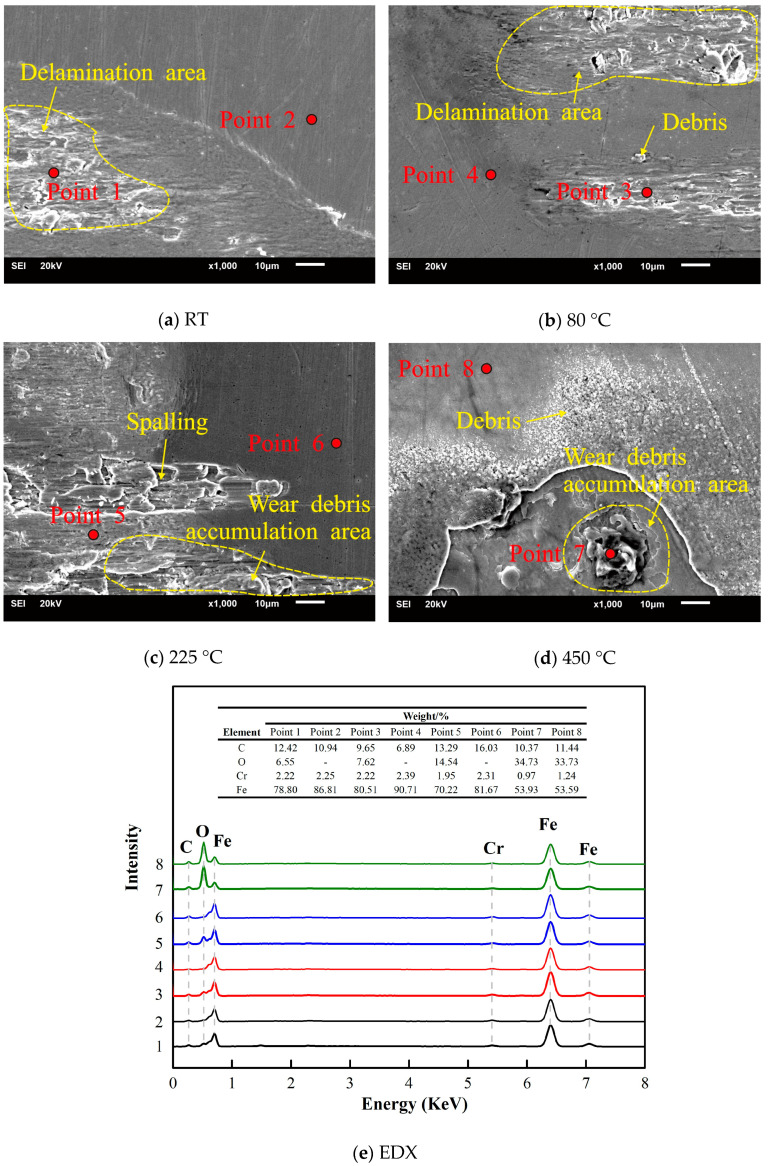
SEM micrographs and EDX of worn scars, D = 50 μm, F = 20 N.

A typical SEM image of the worn scars was observed to study the wear mechanism of 2.25Cr-1Mo steel. As shown in Figure 8, wear debris hardly accumulate but scatter in the concave surface of the friction interface or the edge of the worn scars at RT (Figure 8a). As the temperature increases to 80 °C, the surface of the scratched area clearly shows ploughing due to abrasive wear (Figure 8b). In addition, the friction interface is relatively smooth and shows a wear debris accumulation area at higher temperatures (Figure 8c,d). Figure 8e shows the element content of points 1–8 within Figure 8a–d. The oxygen content of the point in the wear scar increases with the temperature increase. At 450 °C, the presence of oxygen is also detected on the substrate other than the wear scar, which indicates that the high temperature oxidized the material before the test. So, at 10 N, scratching, ploughing, abrasive, and oxidation wear are the main wear mechanisms of the 2.25Cr-1Mo steel tube. As shown in Figure 9, wear debris tend to accumulate at 20 N. At RT and 80 °C, the friction interface displays an obvious delamination area (Figure 9a,b). As the temperature increases to 225 °C and 450 °C, the surface of the scratched area clearly shows spalling and a wear debris accumulation area (Figure 9c,d). Figure 9e shows the element content of points 1–8 within Figure 9a–d. The oxygen content of the point in the wear scar also increases with the temperature increase. Meanwhile, at 450 °C, the presence of oxygen is also detected on the substrate other than the wear scar. This confirms the surface color change in Figure 4. So, at 20 N, spalling, delamination, adhesive, and oxidation wear are the main mechanisms of wear of the 2.25Cr-1Mo steel tube. Besides, in summary, as the temperature increases, the wear mechanism gradually changes from abrasive wear to adhesive wear.

**Figure 10:**
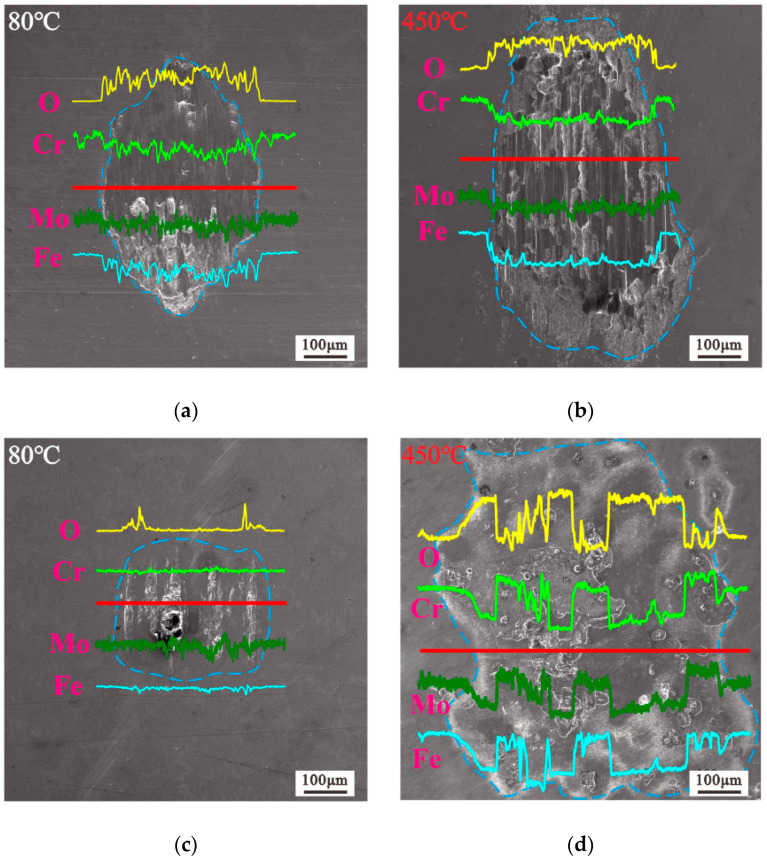
EDX analysis of worn scars, D = 50 μm: (**a**), (**b**) F = 10 N; (**c**), (**d**) F = 20 N.

The amounts of oxygen, chromium, molybdenum, and iron on the wear surface under four test conditions were measured by EDX (the accelerating voltage for EDX is 20 kv). The results are shown in Figure 10. The oxygen content is relatively high on the entire surface of the worn scar, and the contents of the other elements are reduced (Figure 10a,b). As mentioned previously, 10 N causes abrasive wear and covers the entire friction interface. As shown in Figure 10c,d, the oxygen content increases and decreases intermittently, and the other elements decrease and increase correspondingly. Evidently, 20 N causes the middle of the worn scar to stick and the edge of the worn scar to be oxidized. The oxygen content on the surface of the substrate that does not penetrate the worn scar at 450 °C is higher than that at 80 °C. Hence, the high temperature promoted the oxidation of 2.25Cr-1Mo steel before the test in this work. Meanwhile, the change in the amounts of chromium, molybdenum, and iron with oxygen at 450 °C is more obvious than that at 80 °C.

**Figure 11:**
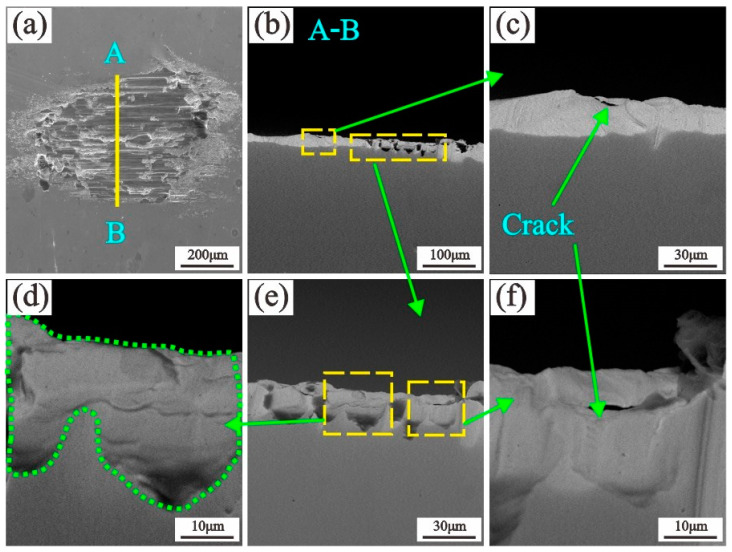
Morphologies of the cross section of a worn scar: T = 450 °C; F = 10 N; D = 50 μm.

The cross section of a sample from test 2 at 450 °C was selected. The sample was cut with an electric spark wire, sanded with sandpaper, and then finally polished with a polishing paste. Section A-B is shown in Figure 11b. Delamination cracks form within the TTS (Figure 11c,f) [38]. Subsurface cracks are caused by surface fatigue effects, and they eventually connect to surface cracks, resulting in spalling and material removal. Meanwhile, the high hardness and refined grains of the TTS layer increase the strength of the surface [39] and may increase the wear resistance of 2.25Cr-1Mo steel.

**Figure 12:**
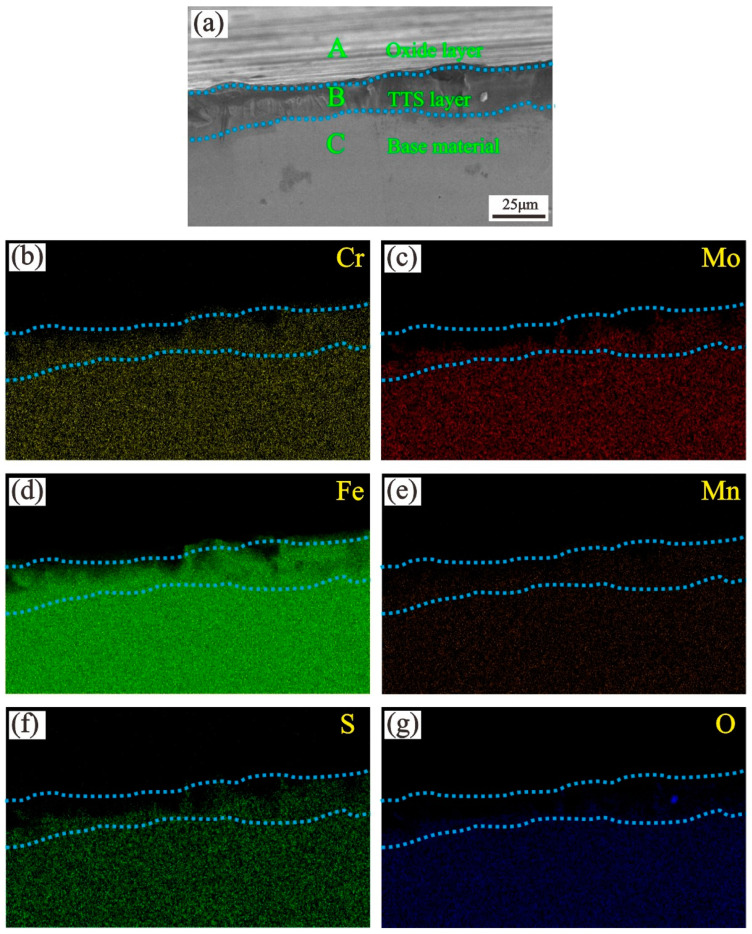
Elemental distribution of the cross section of a worn scar: T = 450 °C; F = 10 N; D = 50 μm.

Figure 12 shows the microstructure and elemental distribution of the portion cross section in Figure 11. Significant stratification morphologies are labeled as A–C. The amounts Cr, Mo, Fe, Mn, and S gradually decrease from the substrate to the oxide layer. In fact, the thickness of the oxide layer is only a few microns and is thus difficult to measure. By contrast, the TTS layer is clearly visible and contains a small amount of oxygen (almost zero) [40] of the same order of the substrate [38].

**Figure 13:**
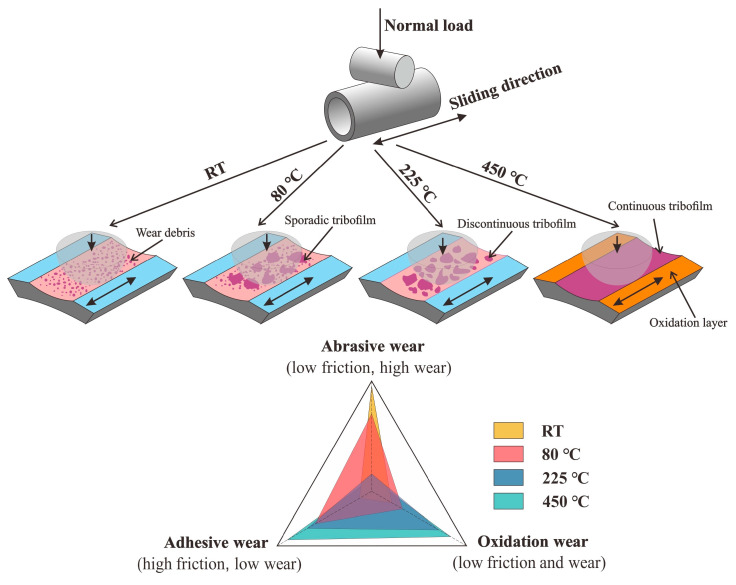
Schematic diagrams of the wear mechanisms.

The wear mechanisms in air can be described by schematic diagrams, as shown in Figure 13. At RT, large wear debris are produced which result in serious abrasive wear and large wear volume. Meanwhile, abrasive debris act as self-lubricating agents [41], resulting in a small COF. At 80 °C, the wear mechanism is mainly the synergy of abrasive wear and adhesive wear, resulting in a larger wear volume and a higher COF. At 225 °C and 450 °C, adhesive wear gradually gains an advantage, resulting in a slight wear. Meanwhile, adhesive wear is influenced by temperature, as increasing temperature led to shorter sliding distances until the onset of severe adhesive wear, distinguished as an increase in the COF [42]. However, the oxide layer that is constantly generated at higher temperatures has the characteristics of an antifriction nature and stabilized high lubrication properties [20]. So, the COF at 450 °C is relatively small. In summary, elevated temperatures will help adhesive wear to defeat abrasive wear, and make the 2.25Cr-1Mo steel show more excellent wear resistance.

## 4. Conclusions

The fretting wear behavior of a 2.25Cr-1Mo heat transfer tube against a Gr5C12 alloy rod with 10^4^ cycles at varied temperatures (including two kinds of normal loads and displacement amplitudes) was studied. There are some other test parameters (such as contact form, tube diameter, test frequency, etc.) that may also affect the wear behavior of the 2.25Cr-1Mo heat transfer tube. This may be studied in the future. On the basis of the abovementioned results, the fretting wear properties of the 2.25Cr-1Mo steel heat transfer tube are described as follows:

(a) Wear volume and COF first increase and then decrease with an increase in temperature. The maximum wear volume and COF occur at 80 °C, whereas the minimum occurs at 450 °C.
(b) Wear mechanisms depend modestly on temperature and largely on normal load. As temperature increases, the wear mechanism gradually changes from abrasive wear to adhesive wear. Scratching, ploughing, abrasive, and oxidation wear are the main wear mechanisms in gross slip conditions at 10 N, whereas spalling, delamination, adhesive, and oxidation wear occur in partial slip conditions at 20 N.
(c) High temperatures (≥225 °C) may improve the wear resistance of 2.25Cr-1Mo steel due to the antifriction nature of the oxidative layer, the high hardness and refined grains of the TTS layer, and extensive material transfer and plastic deformation. This provides data support for the practical engineering application of 2.25Cr-1Mo steel at elevated temperatures.

## Figures and Tables

**Table 1:** Nominal compositions of tribo-pairs in wt.%.

Materials	Element							
	C	Mn	P	S	Si	Cr	Mo	Fe
2.25Cr-1Mo	0.11	0.31	0.007	0.003	0.14	2.42	1.05	bal
Gr5C12	≤0.15	0.30–0.60	≤0.035	≤0.035	≤0.50	4.00–6.00	0.45–0.65	bal

**Table 2:** Experimental parameters for fretting wear tests.

Item	Value
Tube materials	2.25Cr-1Mo
Tube hardness	177.73 HV
Tube Young’s modulus	194.73 Gpa
Rod materials	Gr5C12
Rod hardness	177.43 HV
Rod Young’s modulus	179.32 Gpa
Temperature	RT, 80 °C, 225 °C, 450 °C
Load	10 N, 20 N
Amplitude	50 μm, 100 μm
Frequency	5 Hz
Number of cycles	10^4^ cycles
